# A novel recombinant anti-cluster of differentiation 20 humanized monoclonal antibody (B001) for the treatment of neuromyelitis optica spectrum disorder: a phase 1, multicenter randomized, double-blind trial

**DOI:** 10.3389/fimmu.2026.1676908

**Published:** 2026-04-16

**Authors:** Dongmei Jia, Huabing Wang, Wei Jiang, Yi Shen, Jun Guo, Daidi Zhao, Meini Zhang, Huaxing Meng, Huiru Xue, Yunxing Song, Qin Yao, Ning Xie, Chao Zhang

**Affiliations:** 1 Department of Neurology, Tianjin Neurological Institute, Tianjin Medical University General Hospital, Tianjin, China; 2 Department of Neurology, Beijing Tiantan Hospital, Capital Medical University, Beijing, China; 3 Department of Neurology, Tangdu Hospital, Air Force Medical University, Xi’an, China; 4 Department of Neurology, First Hospital of Shanxi Medical University, Taiyuan, China; 5 Research and Development, Shanghai Pharmaceuticals Holding Co., Ltd., Beijing, China

**Keywords:** B cells, CD20, neuromyelitis optic spectrum disorder, pharmacodynamics, pharmacokinetics

## Abstract

**Introduction:**

B001 is a recombinant humanized anti-CD20 monoclonal antibody targeting CD20+ B cells, that has demonstrated superior B cell depletion and anti-proliferative and cytotoxic effects compared to rituximab in a pre-clinical study. The present phase 1b trial assessed the safety, tolerability, pharmacokinetics, pharmacodynamics, immunogenicity and preliminary efficacy of B001 in aquaporin-4 immunoglobulin G-positive neuromyelitis optica spectrum disorder (NMOSD).

**Methods:**

This phase 1b randomized, double-blind, placebo-controlled trial screened 25 NMOSD patients (April 2022–June 2024). Twenty-two patients received intravenous B001 (350, 700 or 1,000 mg) or placebo via a 3 + 3 dose escalation design, randomized 3:1 to active drug or placebo on days 1 and 15. The primary endpoints were the occurrence of dose-limiting toxicity (DLT) and to recommend the dosage for the phase 2 trial.

**Results:**

Among 22 randomized patients (350 mg: *n* = 3; 700 mg: *n* = 8; 1,000 mg: *n* = 6; placebo: *n* = 5), 20 (90.9%) completed the study. No DLT occurred in evaluable patients. Treatment-related adverse events (TRAEs) occurred in 7/21 (33.3%) patients, including urinary tract infection (14.3%), infusion-related reactions (9.5%), abnormal blood routine tests (9.5%) and hyperlipidemia (4.8%). No TRAEs led to discontinuation, dose reduction or death. Pharmacokinetic analysis revealed that supra-proportional exposure increased at 1,000 mg *vs*. 700 mg. Pharmacodynamics showed sustained B cell depletion (nearly 0/μL) for 24 weeks. No NMOSD relapses occurred during the 24-week study.

**Conclusion:**

B001 demonstrated favorable safety and tolerability, with 700 mg selected as the recommended phase 2 dose.

**Clinical Trial Registration:**

https://clinicaltrials.gov/study/NCT05145361, identifier NCT05145361.

## 1 Introduction

In recent years, the prevalence and annual incidence of neuromyelitis optica spectrum disorder (NMOSD) have been estimated at 1.21 to 1.81 per 1,000 individuals and 0.135 to 0.420 per 1,000, respectively (1). NMOSD is a rare autoimmune neurological disease mediated by B cells, involving complex immune mechanisms including the complement system, B cells and interleukin-6 (IL-6) pathways (2–4). Approximately 90% of NMOSD patients test positive for aquaporin-4 (AQP4) immunoglobulin G (IgG), a critical biomarker in NMOSD diagnosis (5). Therefore, monoclonal antibody therapies targeting AQP4-IgG-positive NMOSD have garnered significant attention. Several FDA-approved medications, including eculizumab, inebilizumab and satralizumab, have shown significant efficacy in preventing relapses of NMOSD (6). Eculizumab, an anti-C5 monoclonal antibody, targets the C5 protein in the complement system, thereby effectively preventing complement-mediated damage to the optic nerves and spinal cord (7). Inebilizumab, an anti-CD19 antibody, suppresses B cell-mediated immune responses by binding to CD19 and promoting B cell depletion. It has shown significant efficacy in reducing NMOSD attacks, slowing disability progression and decreasing hospitalization rates (8), particularly in AQP4 antibody-positive patients (9). Satralizumab inhibits IL-6 signaling by binding to the IL-6 receptor, thereby blocking IL-6-mediated immune damage associated with B cells (10). Since 2020, ofatumumab, an anti-CD20 antibody, has emerged as an important option for treating AQP4-IgG-positive NMOSD, particularly in recurrent and refractory cases (11). However, recent reports have indicated that some patients treated with rituximab experienced breakthrough attacks or adverse reactions, such as serum sickness characterized by arthritis and rash (12). These events may be associated with mechanisms involving complement inhibition, B cell depletion and IL-6 receptor blockade (12). Notably, inebilizumab, which targets CD19+ B cells, has shown promising efficacy in patients switching from rituximab, with no subsequent attacks observed (12). In rare cases of double-positive NMOSD (AQP4 and MOG-IgG), ofatumumab (CD20) has been reported to be an effective alternative treatment option (13). Given the current inadequacy of treatments for NMOSD relapse in the Chinese pharmaceutical market, further investigation and evaluation of novel therapeutic strategies remain urgently needed.

B001 is a recombinant humanized monoclonal antibody targeting CD20, engineered from the murine anti-human CD20 antibody 2B8 and humanized to 96% sequence homology. It was designed to recognize a CD20 epitope distinct from that bound by rituximab, with the aim of enhancing B-cell depletion efficiency while reducing antibody-induced internalization, thereby prolonging its duration of action *in vivo* (14). In contrast, inebilizumab targets CD19 and depletes a broader spectrum of B-cell subsets—including early and mature B cells, plasma blasts, and a subset of plasma cells—resulting in a much wider B-cell suppression profile than that of the CD20-directed B001 (15). Because B001 is structurally closer to a fully human anti-CD20 antibody, its immunogenicity and the risk of related adverse events (AEs; e.g., infections, hypogammaglobulinemia) are theoretically expected to be lower than those associated with earlier chimeric antibodies. Moreover, compared with the broader B-cell depletion induced by inebilizumab, B001 provides more selective targeting of CD20+ B cells, which may confer a more controllable impact on immunoglobulin levels and infection risk (15).

Preclinical studies have demonstrated that B001 significantly enhances survival rates in experimental autoimmune encephalomyelitis models in rhesus macaques, delays disease onset, and improves central nervous system inflammation and demyelination. B001 has completed phase 1a clinical trials, evaluating safety, tolerability, pharmacokinetics (PK) and pharmacodynamics (PD) in relapsed/refractory B cell non-Hodgkin lymphoma (NHL) patients. Preliminary results confirm good clinical safety and tolerability of B001 in NHL patients, demonstrating effective B cell depletion and antitumor activity comparable to early reports of rituximab (16). Based on these preliminary data, this phase 1b clinical study will further investigate the safety, tolerability, PK, PD, immunogenicity and preliminary efficacy of B001 in AQP4-IgG-positive NMOSD patients, providing appropriate dosing guidance for phase 2/3 studies.

## 2 Materials and methods

### 2.1 Study design

#### 2.1.1 Dose escalation

This multicenter, dose escalation clinical trial of B001 was undertaken in patients with NMOSD. A traditional 3 + 3 design was used with 4 to 8 patients per dose level and escalating the dose incrementally until ≥ 2 patients in a dose cohort experienced dose-limiting toxicity (DLT) during the DLT assessment period (from 72 hours after the first dose administration to 72 hours after the second dose administration). The initial dose of B001 was 350 mg. Using a central randomization system (interactive web response system, IWRS), 4 patients were randomized in a ratio of 3:1 to receive either the initial dose of 350 mg B001 or placebo by intravenous infusion on the 1^st^ day (D1) and the 15^th^ day (D15). DLT was defined as any grade ≥ 3 AE according to the Common Terminology Criteria for Adverse Events (CTCAE) version 5.0, except for laboratory test changes without clinical significance thus: 1) if no DLT occurred, escalation to the 700 mg dose level proceeded immediately; 2) if DLT occurred in 1 case out of the initial 4 patients, an additional 4 patients were re-enrolled and randomized in a ratio of 3:1 to receive B001 and placebo. If DLT occurred in ≤ 1 case of the 8 patients, escalation proceeded to the 700 mg dose level; if DLT occurred in ≥ 2 of the 8 patients, 350 mg was considered as an intolerable dose and escalation was discontinued; 3) at the 700 mg and 1,000 mg dose levels, 8 patients were randomized in a ratio of 6:2 to receive B001 and placebo, respectively. The study protocol was reviewed and approved by the Institutional Review Boards of Tianjin Medical University General Hospital (approval No. IRB2021-137-02), Beijing Tiantan Hospital, Capital Medical University (approval No. YW2021-058-04), Tangdu Hospital, Air Force Medical University (approval No. K202209-08), and First Hospital of Shanxi Medical University (approval No. [2021]-Ethical Review-(159)). The trial was carried out in accordance with the principles of the Declaration of Helsinki and the Good Clinical Practice guidelines and was prospectively registered at ClinicalTrials.gov (number NCT05145361, URL: https://clinicaltrials.gov/study/NCT05145361).

Written informed consent was obtained from all patients prior to enrollment in the trial. The route of administration (intravenous infusion) and the dosing schedule specified in the study protocol (two intravenous infusions administered on D1 and D15).

#### 2.1.2 Randomization and blinding

During the dose-escalation phase, patients were randomized in a 3:1 ratio to receive the B001 or placebo group through an IWRS. After providing written the informed consent form and meeting all eligibility criteria, patients received study treatment according to their enrollment order and assigned randomization number. Group allocation information was concealed within the IWRS throughout the study. Patients who withdrew or were withdrawn from the trial retained their randomization numbers and were not allowed to be re-enrolled. Study drug labeling and blinding were performed by personnel independent of the study, drug codes generated by SAS, with all blinding records appropriately maintained. Unblinding at each dose level was performed only after completion and evaluation of DLT assessment, with the unblinding documentation co-signed by the principal investigator, sponsor, and statistician.

### 2.2 Patients

We enrolled patients aged ≥ 18 years old with AP4-IgG-positive NMOSD as defined by the International Panel for NMO Diagnosis criteria of 2015, who had at least 1 relapse during the last 12 months or 2 relapses in the past 2 years prior to screening, using expanded disability status scale (EDSS) scores ≤ 7.5, with negative blood pregnancy tests in fertile females 7 days before the first dose, and with agreement for the use of reliable contraceptive measures for all fertile patients. The key exclusion criteria included: patients with severe NMOSD who may have required ventilatory support; those with immunodeficiency syndromes or chronic active immune system diseases currently undergoing treatment; and patients who had received anti-CD20 therapy or other B cell depletion agents, immunosuppressive therapies or biological agents (such as satralizumab, tocilizumab or eculizumab) within 3 months prior to the first administration of the study dose. Additionally, patients who had been treated with intravenous or intramuscular glucocorticoids, plasmapheresis or intravenous immunoglobulin prior to the first dose were excluded.

### 2.3 Safety and efficacy assessments

Safety was regularly monitored throughout the study period, including vital signs, physical examinations, clinical laboratory tests, 12-lead ECG and AEs. AEs were graded according to the CTCAE version 5.0 and their correlation with the study drug was determined. Relapses of NMOSD were assessed according to clinical symptoms (including acute myelitis, area postrema syndrome, acute brainstem syndrome, acute diencephalic syndrome or hypersomnia, and symptomatic cerebral syndrome) and MRI findings during the double-blind period. Changes in EDSS scores as well as low-contrast visual acuity (LCVA), assessed by the Sloan Low-Contrast Letter Score (SLCLS) at weeks 12 and 24, were compared to baseline.

### 2.4 Immunogenicity evaluation

Blood samples for anti-drug antibody (ADA) were collected at pre-dose on D1, D15, D29, D57, D85 and D169/end of the study or safety visit. Examinations of neutralizing antibody (Nab) were conducted once ADAs were positive.

### 2.5 PK assessments

PK analyses were conducted for patients in all dose groups. Blood samples for PK evaluations were collected prior to dosing on D1, 2 hours after the first administration, immediately and 4 hours after the first administration, D2, D8 and D15 before dosing, 2 hours after the second administration, 2 and 4 hours after the second administration, and on D16, D18, D22, D29, D43, D57, D85, D113 and D169 until the end of the study or the follow-up safety visit.

### 2.6 PD assessments

PD analyses were conducted for patients in all dose groups. Blood samples for PD evaluations were collected at pre-dose on D1, D2 and D8, pre-dose on D15, D16, D29, D57, D85, D113 and D169/end of the study or safety visits. CD19+ B cells in peripheral blood were measured using cytometry and B cell depletion was evaluated.

### 2.7 Biomarker evaluation

Blood samples for biomarker evaluation (mainly AQP4-IgG) were collected at pre-dose on D1, D15, D29, D85 and D169/end of the study or safety visit.

### 2.8 Statistical analysis and sample size

This phase 1b study did not include a formal sample size calculation. Approximately 22–25 participants were enrolled in the dose-escalation phase, and about 20 participants were enrolled in the dose-expansion phase. Safety was assessed in patients who received at least one infusion of the study drug (safety set). Efficacy analysis was based on the full analysis set (FAS) and the efficacy-evaluable set. For patients with NMOSD relapses, the time to the first relapse from enrollment was estimated using the Kaplan-Meier method, including median and quartiles (Q1, Q3). If a patient had not relapsed by the time of withdrawal from the study or the data cutoff, the analysis was censored at these times. Changes in the EDSS scores relative to baseline were also assessed. The number and percentage of patients with and without relapses at week 24 in each dose group were calculated, with the 95% confidence intervals for relapse rates derived using the Clopper-Pearson method. Additionally, a table was provided to describe relapse status, changes in EDSS scores and changes in LCVA as assessed by the SLCLS.

Safety analyses were performed using descriptive statistics. AEs were coded according to the MedDRA terminology and summarized by System Organ Class and Preferred Term, with incidence rates analyzed for treatment-emergent AEs and treatment-related adverse events (TRAEs) by severity. PK characteristics were described using a population PK modeling approach, and PK parameters were calculated using noncompartmental analysis. PD and immunogenicity data were presented descriptively. For patients who tested positive for ADAs, further analyses were conducted to characterize the time of ADA onset, duration, and changes in antibody titters. Evaluation of the exposure–response relationship was performed to further elucidate the association between drug exposure and immunogenicity response.

Because this was an exploratory phase 1b study with a limited sample size, no formal statistical power or sensitivity analyses were conducted. The primary objectives were to assess the safety, tolerability, and preliminary PK/PD profiles of the investigational drug to support dose selection for subsequent phase 2 studies.

All statistical analyses were performed using SAS version 9.4 (SAS Institute Inc., Cary, North Carolina). Baseline and clinical characteristics of patients were summarized using descriptive statistics. Continuous variables are presented with the number of cases, mean, standard deviation, median, quartiles (Q1, Q3), minimum and maximum values. Categorical variables are presented with the number of cases and percentage for each category. Categorical variables were compared using Fisher’s exact test, and continuous variables using the Mann-Whitney U test. Statistical significance was set at a *P*-value < 0.05.

## 3 Results

### 3.1 Patient characteristics and disposition

**Figure 1:**
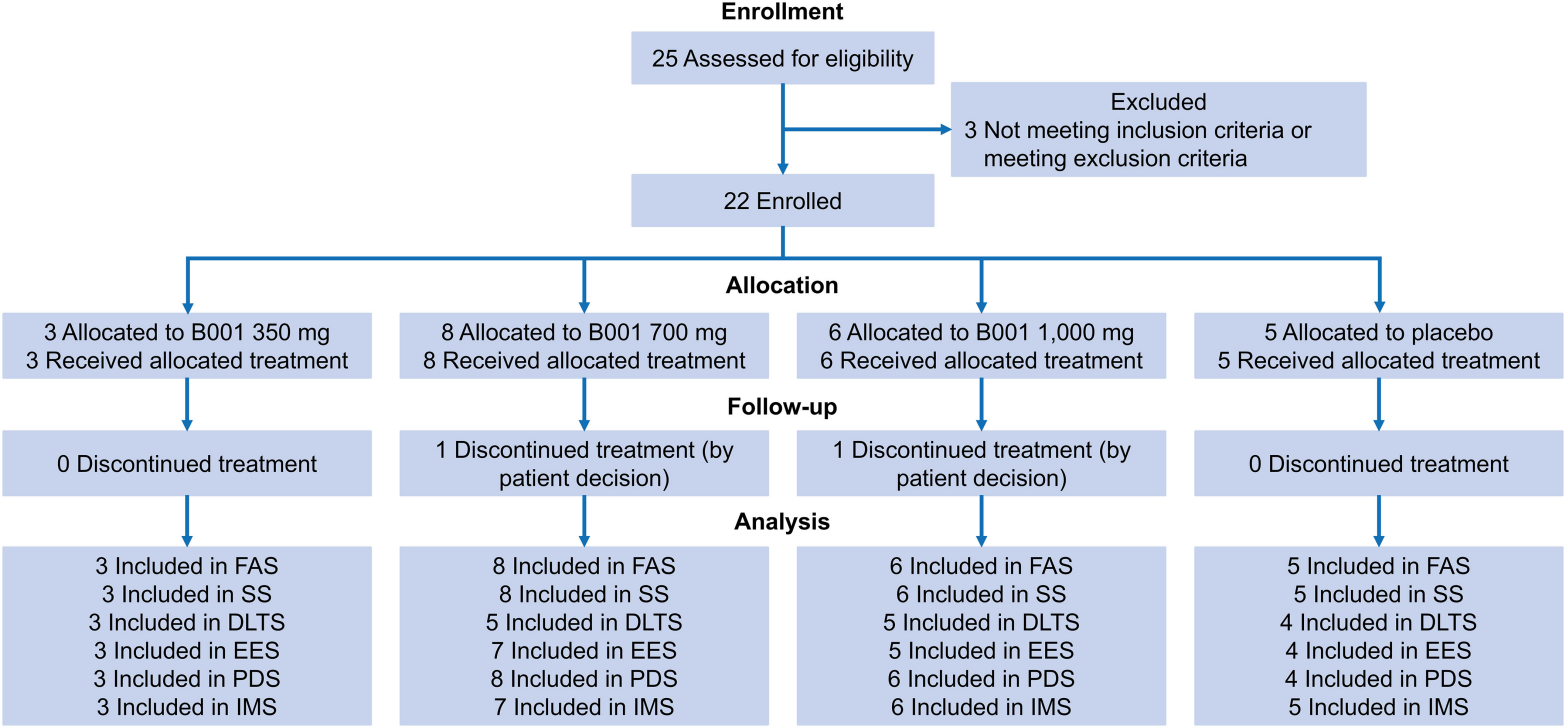
CONSORT flow diagram. DLTS, dose limiting toxicity set; EES, efficacy-evaluable set; FAS, full analysis set; IMS, immunogenicity set; PDS, pharmacodynamics set; SS, safety set.

This study was conducted from April 7, 2022 to June 18, 2024, in four major institutions in China, with a total of 22 patients out of 25 screened enrolled. Of these, 20 (90.9%) completed the study and 2 (9.1%) voluntarily withdrew (**Figure 1**). In the FAS population, the median age was 51.0 (IQR: 35.0, 57.0) years, with 6 (27.3%) males and 16 (72.7%) females. All patients received corticosteroid (glucocorticoid) treatment, 7 had previously received CD20 inhibitors and 6 had been treated with immunosuppressants (**Table 1**). Safety analysis was conducted in 22 patients who had received at least 1 dose of the study drug. There was 1 patient in the placebo group whose safety analysis was analyzed separately due to the individual receiving 1 dose of 200 mg B001 by mistake and hence they were excluded from the safety set. Seven patients had previously received the CD20 inhibitor rituximab, and the interval between the last rituximab administration and the first B001 dose ranged from 9.4 to 43.1 months (**Supplementary Table 1**).

**Table 1:** Baseline demographic and clinical characteristics of patients in each group.

Characteristic	B001			Placebo (*N* = 5)	Total (*N* = 22)
	350 mg (*N* = 3)	700 mg (*N* = 8)	1,000 mg (*N* = 6)		
Demographics					
Age (years), median (IQR)	34.0 (30.0, 43.0)	56.5 (46.0, 67.0)	52.0 (35.0, 57.0)	43.0 (33.0, 52.0)	51.0 (35.0, 57.0)
Sex, n (%)					
Male	0	3 (37.5)	2 (33.3)	1 (20.0)	6 (27.3)
Female	3 (100.0)	5 (62.5)	4 (66.7)	4 (80.0)	16 (72.7)
Height (cm), median (IQR)	160.0 (155.0, 168.0)	160.0 (158.0, 165.0)	164.0 (155.0, 171.0)	165.0 (160.0, 165.0)	160.0 (158.0, 168.0)
Weight (kg), median (IQR)	49.0 (48.0, 65.0)	62.0 (50.5, 67.5)	63.0 (54.0, 81.0)	60.0 (54.0, 62.0)	60.0 (51.0, 66.0)
BMI (kg/cm2), median (IQR)	20.0 (19.1, 23.0)	22.3 (19.6, 25.2)	25.0 (21.1, 27.7)	23.4 (19.5, 24.8)	23.2 (20.0, 25.4)
NMOSD history					
Relapse times, median (IQR)					
Within one year	1.0 (1.0, 1.0)	1.0 (1.0, 1.5)	1.0 (1.0, 1.0)	1.0 (1.0, 1.0)	1.0 (1.0, 1.0)
Within two years	1.0 (1.0, 1.0)	2.0 (2.0, 2.5)	1.0 (1.0, 1.0)	1.0 (1.0, 2.0)	1.0 (1.0, 2.0)
Received treatment, n (%)	3 (100.0)	8 (100.0)	6 (100.0)	5 (100.0)	22 (100.0)
Corticosteroids (Glucocorticoids)	3 (100.0)	8 (100.0)	6 (100.0)	5 (100.0)	22 (100.0)
CD20 inhibitor	1 (33.3)	3 (37.5)	1 (16.7)	2 (40.0)	7 (31.8)
Immunosuppressants	1 (33.3)	3 (37.5)	1 (16.7)	1 (20.0)	6 (27.3)
Immune serum globulin	0 (0.0)	4 (50.0)	0 (0.0)	1 (20.0)	5 (22.7)
Other therapies	0 (0.0)	1 (12.5)	1 (16.7)	0 (0.0)	2 (9.1)
Unspecified herbs and traditional medicines	0 (0.0)	1 (12.5)	0 (0.0)	0 (0.0)	1 (4.5)

NMOSD, neuromyelitis optica spectrum disorder.

### 3.2 Safety and tolerability

The median duration of treatment exposure was 15 days across all groups, with a consistent median relative dose intensity of 100.0% (IQR: 100.0%, 100.0%) for all the B001 groups. No dose adjustments were required during the study for any enrolled patients. During the study, no DLT event occurred in 17 evaluable patients. TRAEs occurred in 7 patients (33.3%), including urinary tract infection (*n =* 3, 14.3%), infusion-related reactions (*n =* 2, 9.5%), abnormal blood routine tests (*n =* 2, 9.5%) and hyperlipidemia (*n =* 1, 4.8%) There were no permanent treatment discontinuations, dosage reductions of the investigational drug, study withdrawals, or deaths due to TRAEs. Temporary discontinuation of administration due to TRAE occurred in 2 (1 in 1,000 mg and 1 in placebo groups) patients (**Table 2**). Grade ≥ 3 anemia was observed in 1 patient in the 350 mg group, also unrelated to B001. No correlation between decreased immunoglobin levels and infection was observed in the 3 patients (1 each in the B001–350 mg, B001–700 mg and placebo group) who experienced investigational drug-related urinary tract infection.

**Table 2:** Incidence of TRAEs that occurred during the study.

TRAE	B001			Placebo (*N =* 4)	Total (*N =* 21)
	350 mg (*N =* 3)	700 mg (*N =* 8)	1,000 mg (*N =* 6)		
Any TRAE	1 (33.3)	1 (12.5)	3 (50.0)	2 (50.0)	7 (33.3)
TRAE with CTCAE grade ≥ 3	0 (0.0)	0 (0.0)	0 (0.0)	0 (0.0)	0 (0.0)
TRAE leading to treatment interruptions	0 (0.0)	0 (0.0)	1 (16.7)	1 (25.0)	2 (9.5)
Urinary tract infection	1 (33.3)	1 (12.5)	0 (0.0)	1 (25.0)	3 (14.3)
Infusion-related reaction	0 (0.0)	0 (0.0)	1 (16.7)	1 (25.0)	2 (9.5)
Leukocyte count decreased	0 (0.0)	0 (0.0)	1 (16.7)	0 (0.0)	1 (4.8)
Lymphocyte percentage decreased	0 (0.0)	0 (0.0)	1 (16.7)	0 (0.0)	1 (4.8)
Lymphocyte count decreased	0 (0.0)	0 (0.0)	1 (16.7)	0 (0.0)	1 (4.8)
Neutropenia	0 (0.0)	0 (0.0)	1 (16.7)	0 (0.0)	1 (4.8)
Hyperlipidaemia	0 (0.0)	0 (0.0)	0 (0.0)	1 (25.0)	1 (4.8)

Data are presented as number of patients (frequency).

CTCAE, Common Terminology Criteria for Adverse Events; TRAE, treatment-related adverse event.

### 3.3 Preliminary efficacy

No relapses of NMOSD occurred in any of the patients during the 24-week study period. The median change in EDSS from baseline at D169/end of study was 0.0 for the 350 mg, 700 mg, and 1,000 mg, with no significant difference compared to the placebo group, indicating minimal neurological disability (**Table 3**). All groups showed no obvious changes from baseline in LCVA as assessed by SLCLS.

**Table 3:** Preliminary efficacy results.

Assessment	B001			Placebo (*N =* 5)	Total (*N =* 22)
	350 mg (*N =* 3)	700 mg (*N =* 8)	1,000 mg (*N =* 6)		
Relapse situation of NMOSD, *n* (%)					
No relapse during trial	3 (100.0)	8 (100.0)	6 (100.0)	5 (100.0)	22 (100.0)
Changes of EDSS score compared to baseline, median (range) [number of patients]					
Day 29	0.0 (0.0, 1.0) [*n =* 3]	-0.3 (-0.5, 0.0) [*n =* 6]	0.0 (-0.5, 0.5) [*n =* 5]	0.0 (-1.0, 0.0) [*n =* 5]	0.0 (-1.0, 0.0) [*n =* 19]
Day 57	0.0 (0.0, 0.0) [*n =* 1]	-0.5 (-1.5, 0.0) [*n =* 6]	0.0 (-1.0, 0.0) [*n =* 5]	0.0 (-1.5, 0.0) [*n =* 5]	0.0 (-1.5, 0.0) [*n =* 17]
Day 85	0.0 (0.0, 0.0) [*n =* 2]	-0.5 (-2.0, 0.0) [*n =* 6]	0.0 (-1.5, 0.0) [*n =* 5]	0.0 (-1.5, 0.0) [*n =* 5]	0.0 (-2.0, 0.0) [*n =* 18]
Day 113	0.0 (0.0, 0.0) [*n =* 1]	-0.5 (-2.0, 0.0) [*n =* 6]	0.0 (-1.5, 0.0) [*n =* 5]	0.0 (-1.0, 0.0) [*n =* 4]	0.0 (-2.0, 0.0) [*n =* 16]
Day 169	0.0 (0.0, 0.0) [*n =* 3]	-1.0 (-3.0, 0.0) [*n =* 7]	0.0 (-2.0, 0.0) [*n =* 5]	0.0 (-1.0, 0.0) [*n =* 4]	0.0 (-3.0, 0.0) [*n =* 19]

EDSS, expanded disability status scale; NMOSD, neuromyelitis optica spectrum disorder.

### 3.4 Pharmacokinetics/Pharmacodynamics

PK analysis of B001 monotherapy was based on blood samples obtained from all 17 patients. Samples were collected on D1 for 350, 700, 1,000 mg and D15 following single-dose administration. These samples were used to evaluate the PK parameters of B001 and the plasma concentration-time profiles for single-dose as shown in **Supplementary Figure 1**.

The median time to reach maximum concentration of the 1^st^ dosing at 350 mg, 700 mg and 1,000 mg B001 was 6.0 hours, 4.2 hours and 4.9 hours, respectively. The area under the concentration–time curve from time zero to the last quantifiable concentration (AUC_0-last_) geometric mean ratio of the 1^st^ dosing and the 2^nd^ dosing of B001 at 350 mg, 700 mg and 1,000 mg was 1:1.9:3.2 and 1:2.0:3.5, respectively. The sum of AUC_0-last_ geometric mean ratios after administration of B001 twice at 350 mg, 700 mg and 1,000 mg were 1:1.9:3.4, showing that the proportion of the increased exposure was significantly higher than that of the escalated dose from 700 mg to 1,000 mg (3.4 *vs*. 2.9). The geometric mean of elimination half-life was 14.6 days, 18.2 days and 22.4 days for 350 mg, 700 mg and 1,000 mg, respectively, suggesting a tendency to increase in a dose-dependent manner (**Table 4**).

**Table 4:** Summary of pharmacokinetic parameters following B001 intravenous infusion.

PK parameters	350 mg (*N* = 3)		700 mg (*N* = 7)		1,000 mg (*N* = 5)	
	*n*	Statistics	*n*	Statistics	*n*	Statistics
After 1^st^ infusion (Day 1)						
C_max_ (μg/mL)	3	209 (24.8%)	7	330 (15.0%)	5	529 (41.2%)
T_max_ (h)	3	6.0 (3.7, 7.4)	7	4.2 (3.6, 8.2)	5	4.9 (4.7, 5.1)
T_last_ (d)	3	14.0 (13.8, 14.0)	7	14.0 (13.9, 14.1)	5	14.1 (14.0, 16.8)
AUC_0-last_ (μg·h/mL)	3	27,112 (31.7%)	7	50,144 (16.8%)	5	85,388 (39.9%)
AUC_0-inf_ (μg·h/mL)	1	23,424	0	–	0	–
AUC_0-14d_ (μg·h/mL)	3	27,101 (31.7%)	7	50,110 (16.8%)	5	82,447 (40.6%)
CL (mL/h)	1	14.9	0	–	0	–
V_z_ (L)	1	2.8	0	–	0	–
t_1/2_ (d)	1	5.3	0	–	0	–
λ_z_ (1/h)	1	0.00544	0	–	0	–
%AUC_ex_ (%)	1	18.9	0	–	0	–
After 2^nd^ infusion (Day 15)						
C_max_ (μg/mL)	3	231 (23.2%)	7	391 (14.5%)	3	629 (52.6%)
T_max_ (h)	3	4.9 (3.4, 4.9)	7	4.2 (4.1, 8.0)	3	5.1 (5.0, 6.8)
T_last_ (d)	1	161.0	6	153.5 (98.0, 161.8)	3	153.8 (150.8, 153.9)
AUC_0-last_ (μg·h/mL)	1	85,156	6	148,791 (15.8%)	3	298,010 (61.3%)
AUC_0-14d_ (μg·h/mL)	3	38,509 (18.9%)	7	70,053 (13.5%)	3	115,416 (52.1%)
CL (mL/h)	0	–	0	–	0	–
V_z_ (L)	0	–	0	–	0	–
t_1/2_ (d)	3	14.6 (24.4%)	7	18.2 (24.0%)	3	22.4 (19.1%)
λ_z_ (1/h)	3	0.00198 (24.4%)	7	0.00158 (24.0%)	3	0.00129 (19.1%)
%AUC_ex_ (%)	3	0.6 (226.6%)	7	0.4 (139.2%)	3	0.8 (110.6%)
Rac_C_max_	3	1.1 (2.7%)	7	1.2 (3.8%)	3	1.1 (8.6%)
Rac_AUC_0-14d_	3	1.4 (15.0%)	7	1.4 (9.0%)	3	1.3 (11.2%)

T_max_ and T_last_ are summarized as median (range), whereas other parameters are summarized as the geometric mean (geometric coefficient of variation, %). When *n* = 2, T_max_ and T_last_ are summarized as median (range), and other parameters are summarized as arithmetic mean (range); When *n* = 1, only individual parameter values are listed.

AUC_0–14d_, area under the concentration–time curve from time zero to 14 days post-dose; AUC_0–inf_, area under the concentration–time curve from time zero to infinity; AUC_0–last_, area under the concentration–time curve from time zero to the last quantifiable concentration; %AUC_ex_, percentage of AUC extrapolated beyond the last quantifiable concentration; CL, clearance; C_max_, maximum plasma concentration; λ_z_, elimination rate constant; Rac_AUC_0–14d_, accumulation ratio based on AUC_0–14d_; Rac_C_max_, accumulation ratio based on C_max_; T_last_, time of the last quantifiable concentration; T_max_, time to reach maximum concentration; t_1/2_, elimination half-life; V_z_, volume of distribution; “–”, Not calculable.

**Figure 2:**
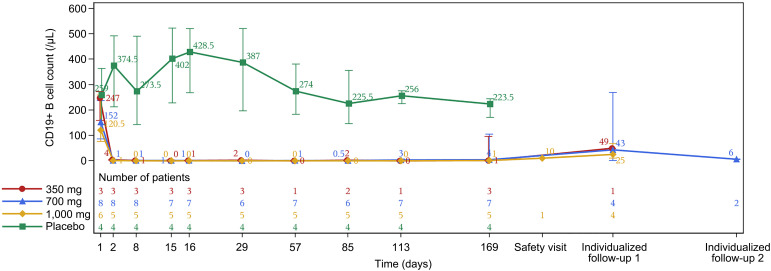
CD19+ B cell counts over time. Data are presented as median (range).

The degree of depletion of B cells was measured in 21 patients. The mean change (SD) of the CD19+ B cell count from baseline decreased rapidly to the minimal level on the first day after administration [-222.3 (60.0), -176.6 (128.3) and-124.0 (82.1)/µL in the 350-, 700- and 1,000-mg groups, respectively], which slightly rebounded at the end of the study (D169) [-193.3 (67.4), -127.1 (168.1) and -110.2 (54.0)/µL in the 350-, 700- and 1,000-mg groups, respectively]. The CD19+ B cell counts in the placebo group fluctuated at a high level without an obvious reduction in the range of 207.5–394.3/µL throughout the study period (**Figure 2**). In addition, there was no significant difference in serum anti-AQP4 antibodies titers in the B001 and placebo groups.

**Figure 3:**
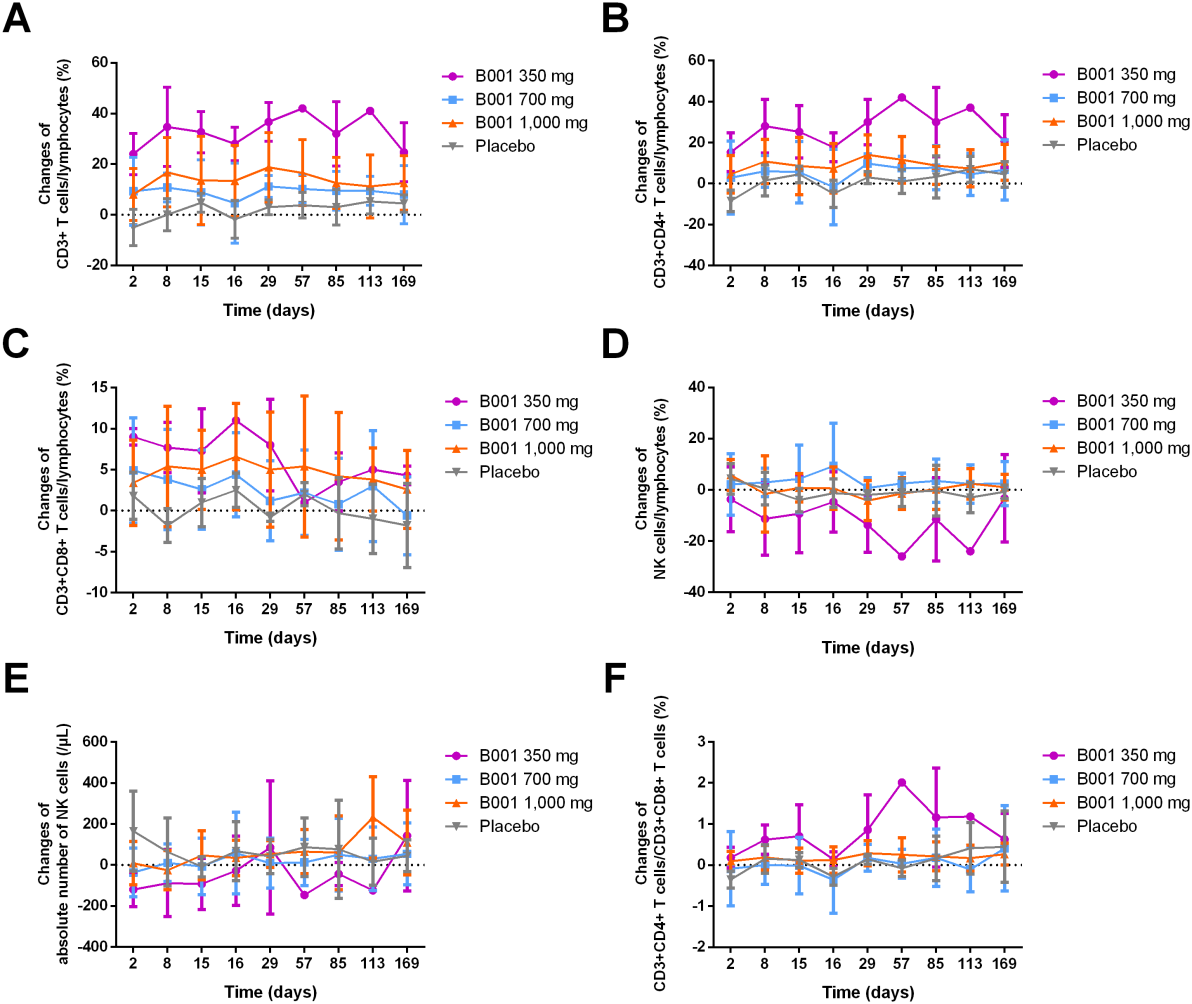
Changes from baseline in B cell–mediated immune responses during the treatment period. **(A)** Change in CD3+ T cells/lymphocytes (%). **(B)** Change in CD3+CD4+ T cells/lymphocytes (%). **(C)** Change in CD3+CD8+ T cells/lymphocytes (%). **(D)** Change in NK cells/lymphocytes (%). **(E)** Change in absolute NK cell count (/μL). **(F)** Change in the CD3+CD4+ T cells/CD3+CD8+ T cells (%). Data in all panels are presented as mean ± SD.

In addition to its direct effect on B cells, B001 also appeared to indirectly influence other immune cells. Compared with placebo, the percentages of CD3+ T cells, CD3+CD4+ T cells and CD3+CD8+ T cells among lymphocytes from baseline were increased after the administration of the three different doses of B001 (**Figures 3A–C**). The proportion of natural killer (NK) cells among lymphocytes relative to baseline in the B001 groups decreased in comparison to the placebo (**Figure 3D**). However, substantial changes were not found for either the absolute count of NK cells or the proportion of CD3+CD4+ T cells relative to CD3+CD8+ T cells from baseline in comparison to placebo (**Figures 3E, F**). Notably, the 700 mg dose appeared to have a relatively smaller impact on T cells compared to the 350 mg and 1,000 mg doses (**Figure 3**).

### 3.5 Immunogenicity

Immunogenicity was examined in 21 patients. ADA was detected positive in 2 patients (1 in the 700 mg group and 1 in the placebo group) at baseline. After administration, ADA was detected positive in 5 (71.4%) patients and 1 (20.0%) patient in the 700 mg and placebo group, respectively (**Supplementary Table 2**). One patient (4003) with a high titer of ADA in the 700 mg group was also Nab positive (**Supplementary Table 3**). In addition, within the dosing regimen and 24-week follow-up period of this trial, B001 did not cause a clinically meaningful decline in immunoglobulin levels (**Supplementary Table 4**). No new-onset abnormal IgG reductions were observed in any dose cohort. Only one participant in the 1000 mg cohort (02008) developed a new-onset decrease in immunoglobulin M (IgM; normal at baseline but below the reference range post-treatment), without any associated infections or invasive disease. Although infection or infestation events were reported in 3/3, 4/8, and 3/6 patients in the 350 mg, 700 mg, and 1,000 mg cohorts, respectively, none of these events were associated with new-onset abnormalities in immunoglobulin levels.

## 4 Discussion

The present phase 1b study evaluated the safety, tolerability, immunogenicity, PK, PD and preliminary efficacy of B001 in patients with AQP4-IgG positive NMOSD. The results demonstrated that B001 was generally safe and well tolerated, with PD responses characterized by rapid and sustained B-cell depletion, indicating favorable pharmacological potential, no DLTs were observed and the 700 mg dose was identified as the recommended phase 2 dose.

B cells play a central role in NMOSD pathogenesis by producing AQP4-IgG, secreting proinflammatory cytokines, and activating autoreactive T cells (17). Rituximab, the first chimeric anti-CD20 monoclonal antibody, has been used to prevent relapses in NMOSD. Prior phase 1 study in relapsed B-cell lymphomas showed rapid B-cell depletion following rituximab administration, with recovery occurring over 3–6 months (16). In relapsed/refractory B-cell NHL patients, B001 demonstrated comparable safety and B-cell depletion activity in a phase 1 study. Furthermore, in NMOSD patients, the humanized anti-CD19 monoclonal antibody inebilizumab significantly reduced B-cell counts within 8 days of 300 mg administration and maintained depletion for up to 197 days (8), leading to its FDA approval in 2020 for relapse prevention in AQP4-IgG–positive NMOSD.

In the present study, B001 at doses of 300–1,000 mg reduced peripheral CD19+ B-cell counts to depletion levels within 2 days, with sustained depletion lasting up to 169 days, demonstrating a potent and durable B-cell–depleting effect comparable to inebilizumab (17). Notably, no significant differences were observed in CD19+ B-cell levels between inebilizumab and rituximab groups, both exhibiting deep B-cell depletion, which may explain the absence of intergroup differences in B-cell counts (18).

The primary PD endpoint was the change in peripheral CD19+ B-cell counts relative to baseline. To ensure reliable assessment, enrollment excluded individuals who had received anti-CD20 or other lymphocyte-depleting therapies within 3 months prior to first dosing, as well as those with baseline CD19+ B-cell counts below the normal range. This ensured that even participants with prior rituximab exposure had fully recovered CD19+ B-cell counts before B001 administration. Seven patients with prior CD20 depletion (all rituximab) had their last dose administered more than 3 months before B001 initiation (20.9, 12.1, 21.8, 13.9, 9.4, 12.3, and 43.1 months; **Supplementary Table 1**), providing sufficient time for CD19+ B-cell recovery.

Recent studies indicate that, besides B cells, other immune cell types contribute to NMOSD pathogenesis. AQP4 antigen stimulation skews immune responses toward Th17, enhancing the release of proinflammatory cytokines such as IL-6 and IL-21 (19). Marino et al. reported that B-cell depletion in mice inhibited alloreactive CD4+ memory T-cell formation while enhancing CD8+ memory T-cell responses (20). Yang et al. found that T-cell subsets and innate immune cells correlated with disease activity in NMOSD, with acute-phase patients showing decreased CD3+ and CD4+ Th cells, and remission-phase patients exhibiting elevated CD8+ T cells and a decreased CD4+/CD8+ ratio (21). In the present study, the proportions of CD3+, CD3+CD4+, and CD3+CD8+ T cells were significantly increased compared with baseline. However, due to the small sample size and baseline differences across dose groups, further classification of T-cell subsets was not feasible, and the specific effects of B001 on T-cell subpopulations could not be fully assessed. Although B001 primarily targets B cells, its indirect modulation of other immune cells remains relevant (22).

Regarding immunoglobulins, in the 700 mg cohort, mean IgG levels at baseline and follow-up visits (D1, D29, D57, D85, D113, and D169/end-of-study) were 705.0, 855.7, 817.0, 916.3, 884.1, and 1,007.4 mg/dL, respectively, and IgM levels were 99.3, 67.3, 68.6, 70.6, 74.8, and 82.9 mg/dL. These results indicate that IgG levels did not decrease post-dose, with slight increases, while IgM levels declined, consistent with B001’s potent anti-CD20 activity. In the 1,000 mg cohort, one participant (02008) experienced a clinically meaningful IgM decrease post-dose without associated infection or invasive disease. No new IgG abnormalities were observed across dose groups. Although infections or invasive events were reported in some participants (350 mg: 3/3; 700 mg: 4/8; 1,000 mg: 3/6), these were not associated with immunoglobulin declines, suggesting no clear correlation between Ig level reductions and infection events. Given the 24-week treatment duration, further studies with larger sample sizes and longer follow-up are needed to assess B001’s impact on immunoglobulins and infection risk.

No new safety signals were observed. No DLTs occurred, and no patients discontinued or died due to TRAEs. Infusion-related reactions occurred in 5.9% of B001 patients (1 patient, 1,000 mg), markedly lower than reported for rituximab or inebilizumab, potentially attributable to B001’s high humanization and reduced complement-dependent cytotoxicity activity (22–24).

The most common AEs with inebilizumab in NMOSD patients were urinary tract infections (26%) and nasopharyngitis (21%) (8); in this study, 2 B001 patients (11.8%) developed urinary tract infections, not statistically different from placebo (1 patient, 20%), supporting further evaluation in larger populations.

B001 PK exhibited dose dependence, with geometric mean half-lives ranging from 14.6 to 22.4 days and slightly greater-than-dose-proportional exposure; immunogenicity had no significant impact on PK parameters. ADA positivity in the 700 mg cohort was 71.4%, versus 20.0% in placebo. Among B001 ADA-positive patients, 80% (4/5) were detected at the last visit (D169), with only one patient showing high titers and confirmed NAb.

The observed higher ADA incidence compared to previous reports may be attributed to subject characteristics in our study, including the exclusively Chinese population with its distinct genetic background, underlying disease status and potential immune sensitization from prior anti-CD20 exposure (25, 26). ADAs may accelerate drug clearance via immune complex formation, while Nabs may compromise drug activity and target engagement. These mechanisms could collectively contribute to diminished therapeutic efficacy and potentially elevate risks of immune complex-mediated AEs like vasculitis or rash (27–29). Nevertheless, in this phase 1b trial, ADA demonstrated no significant effect on PK/PD or efficacy. The clinical relevance these findings therefore remains uncertain in our limited cohort, meriting further investigation in expanded clinical studies with longer follow-up.

Comparative evidence from established anti-CD20 therapies reveals substantially lower immunogenicity profiles. Ocrelizumab and ofatumumab both exhibit minimal ADA incidence, attributed to their advanced humanization profiles. Ofatumumab maintains ADA rates are < 1%, with rare neutralization and negligible impact on drug exposure or efficacy (30). Similarly, OPERA I/II studies reported baseline ocrelizumab ADA incidence of 0.3–1.0% and treatment-emergent rates of 0.2–0.5%, with ~85–90% humanization identified as a critical factor underlying this low immunogenicity (31, 32). Overall, next-generation anti-CD20 antibodies are less likely to induce ADA and are better tolerated than first-generation rituximab.

Although this represents the first systematic early clinical evaluation of B001 in NMOSD, early-phase data for CD20/CD19 antibodies in AQP4-IgG–positive NMOSD remain limited, with most evidence derived from phase 2/3 randomized trials (8, 21, 33, 34). Therefore, observed efficacy signals should be considered exploratory rather than confirmatory.

Limitations include a relatively small sample size (*n* = 22) and 24-week follow-up, restricting the evaluation of long-term efficacy, relapse rates, and low-frequency AEs. Additionally, the current sample size does not permit robust subgroup analyses by demographic factors such as sex and age. These analyses are planned for subsequent phase 2/3 trials, where increased power will support meaningful investigation of such variables. No NMOSD relapses occurred during the study, indicating that B001 effectively depletes B cells without triggering disease relapses, even under sustained immune activity. Considering the central role of B cells in NMOSD pathogenesis, B001 has potential as a disease-modifying therapy; however, larger studies are needed to assess long-term effects on relapse rate and visual outcomes.

The present trial found that none of the patients experienced NMOSD relapses during the 24-week study period. Given the central role of B cells in the pathogenesis of NMOSD, it was encouraging to find that B001 effectively depleted B cells without triggering disease relapse, even in patients with ongoing immunologic activity. These findings suggest that B001 may offer significant potential as a disease-modifying therapy for NMOSD, especially in patients with AQP4-IgG antibodies who are at a high risk of disease relapse. However, we did not observe significant changes in EDSS scores, vision and the rate of relapse probably due to the small sample size and short study period. A larger scale study is needed to evaluate the impact of B001 on the clinical outcomes of recurrent NMOSD. Taken together, the results from the present trial have shown that B001 has an acceptable safety profile and produced preliminary signs of clinical activity in NMOSD, thereby warranting further clinical studies.

## Data Availability

The raw data supporting the conclusions of this article will be made available by the authors, without undue reservation.
