# Supplementary material for: A novel recombinant anti-cluster of differentiation 20 humanized monoclonal antibody (B001) for the treatment of neuromyelitis optica spectrum disorder: a phase 1, multicenter randomized, double-blind trial
Source: Front Immunol. 2026 Apr 16;17:1676908. doi: 10.3389/fimmu.2026.1676908 (PMC13128562; doi:10.3389/fimmu.2026.1676908)
Supplement: Supplementary file 1 [file DataSheet1.docx]

Supplementary Materials

**TABLE S1 Dosing frequency.**

| Dosage | Patient code | Time interval between last rituximab administration and first B001 dose (months) | Rituximab dose | Rituximab dosing frequency |
| --- | --- | --- | --- | --- |
| B001 350 mg | 02003 | 20.9 | 500 mg | 5 |
| B001 700 mg | 01003 | 12.1 | 100 mg | 2 |
|  | 01005 | 21.8 | 100 mg | 2 |
|  | 02002 | 13.9 | 500 mg | 2 |
| B001 1,000 mg | 04002 | 9.4 | 100 mg | 5 |
| Placebo | 01004 | 12.3 | 100 mg | 4 |
|  | 04001 | 43.1 | 100 mg | 3 |

**TABLE S2 Examination of ADA and Nab in the study groups.**

| Visit | Examination | B001 | | | Placebo  (*N =* 5) | Total  (*N =* 21) |
| --- | --- | --- | --- | --- | --- | --- |
|  |  | 350 mg  (*N =* 3) | 700 mg  (*N =* 7) | 1,000 mg  (*N =* 6) |  |  |
| Baseline | ADA |  |  |  |  |  |
|  | Positive | 0 (0.0) | 1 (14.3) | 0 (0.0) | 1 (20.0) | 2 (9.5) |
|  | Negative | 3 (100.0) | 6 (85.7) | 6 (100.0) | 4 (80.0) | 19 (90.5) |
|  | Nab |  |  |  |  |  |
|  | Positive | 0 (0.0) | 0 (0.0) | 0 (0.0) | 0 (0.0) | 0 (0.0) |
|  | Negative | 0 (0.0) | 1 (100.0) | 0 (0.0) | 1 (100.0) | 2 (100.0) |
| Post-administration | ADA |  |  |  |  |  |
|  | Positive | 0 (0.0) | 5 (71.4) | 0 (0.0) | 1 (20.0) | 6 (28.6) |
|  | Negative | 3 (100.0) | 2 (28.6) | 6 (100.0) | 4 (80.0) | 15 (71.4) |
|  | Nab |  |  |  |  |  |
|  | Positive | 0 (0.0) | 1 (20.0) | 0 (0.0) | 0 (0.0) | 1 (16.7) |
|  | Negative | 0 (0.0) | 4 (80.0) | 0 (0.0) | 1 (100.0) | 5 (83.3) |

Note: Data are presented as number of patients (percentage).

Abbreviations: ADA, anti-drug antibody; Nab, neutralizing antibody.

**TABLE S3 Titers of ADA-positive patients.**

| Group | Patient code | Visit (Day) | Titer (dilution fold) |
| --- | --- | --- | --- |
| 700 mg | 1003 | 2 (1) | 26.34 |
|  |  | 9 (29) | 1.42 |
|  | 1005 | 14 (169) | 1 |
|  | 2004 | 14 (169) | 3.18 |
|  | 4003 | 14 (169) | 74.45 |
|  | 4004 | 14 (169) | 2.87 |
| Placebo | 1004 | 2 (1) | 1.16 |
|  |  | 5 (15) | 1 |

Abbreviation: ADA, anti-drug antibody.

**TABLE S4** **Changes in immunoglobulin levels relative to baseline.**

|  | B001 | | | Placebo  (*N =* 5) | Total  (*N =* 22) |
| --- | --- | --- | --- | --- | --- |
|  | 350 mg  (*N =* 3) | 700 mg  (*N =* 8) | 1,000 mg  (*N =* 6) |  |  |
| IgM (mg/dL), mean ± SD | | | | | |
| Baseline | 89.0 ± 49.7 | 79.9 ± 34.6 | 82.5 ± 23.9 | 86.3 ± 28.9 | 83.3 ± 30.7 |
| Relative change from baseline |  |  |  |  |  |
| D1 | 21.0 ± - | -16.4 ± 30.3 | - ± - | -3.6 ± - | -6.3 ± 26.9 |
| D29 | -5.7 ± 5.0 | -9.6 ± 27.2 | -5.2 ± 16.0 | 1.2 ± 16.6 | -5.2 ± 19.1 |
| D57 | -21.9 ± 2.0 | -12.4 ± 30.7 | -17.2 ± 20.1 | -0.5 ± 16.2 | -11.5 ± 22.2 |
| D85 | -17.7 ± 12.7 | -6.3 ± 25.0 | -16.0 ± 29.8 | -3.1 ± 17.2 | -9.6 ± 22.4 |
| D113 | -29.1 ± 4.2 | -2.0 ± 37.9 | -19.0 ± 27.0 | 0.7 ± 13.8 | -8.6 ± 28.3 |
| D169 | -13.8 ± 11.2 | 6.0 ± 40.8 | -20.4 ± 24.4 | -3.6 ± 24.5 | -6.0 ± 30.2 |
| IgG (mg/dL), mean ± SD | | | | | |
| Baseline | 898.3 ± 72.5 | 832.1 ± 118.7 | 726.7 ± 196.3 | 863.8 ± 170.0 | 819.6 ± 154.1 |
| Relative change from baseline |  |  |  |  |  |
| D1 | 178.0 ± - | -44.0 ± 91.6 | - ± - | -60.0 ± - | -2.8 ± 120.2 |
| D29 | 37.3 ± 45.6 | 21.9 ± 62.4 | 87.2 ± 119.4 | 49.6 ± 139.4 | 47.5 ±96.0 |
| D57 | -61.6 ± 43.1 | 17.5 ± 79.9 | 20.4 ± 109.3 | 85.2 ± 103.4 | 28.3 ± 96.3 |
| D85 | 99.3 ± 127.6 | 82.4 ± 100.3 | 17.8 ± 138.8 | 89.2 ± 115.5 | 70.5 ± 113.0 |
| D113 | -69.2 ± 17.2 | 50.3 ± 147.0 | 48.8 ± 129.6 | 62.8 ± 87.1 | 40.6 ± 119.0 |
| D169 | 252.0 ± 192.8 | 173.6 ± 251.1 | 45.2 ± 168.1 | 174.8 ± 142.9 | 153.6 ± 197.4 |

Abbreviations: D, day; IgG, immunoglobulin G; IgM, immunoglobulin M.


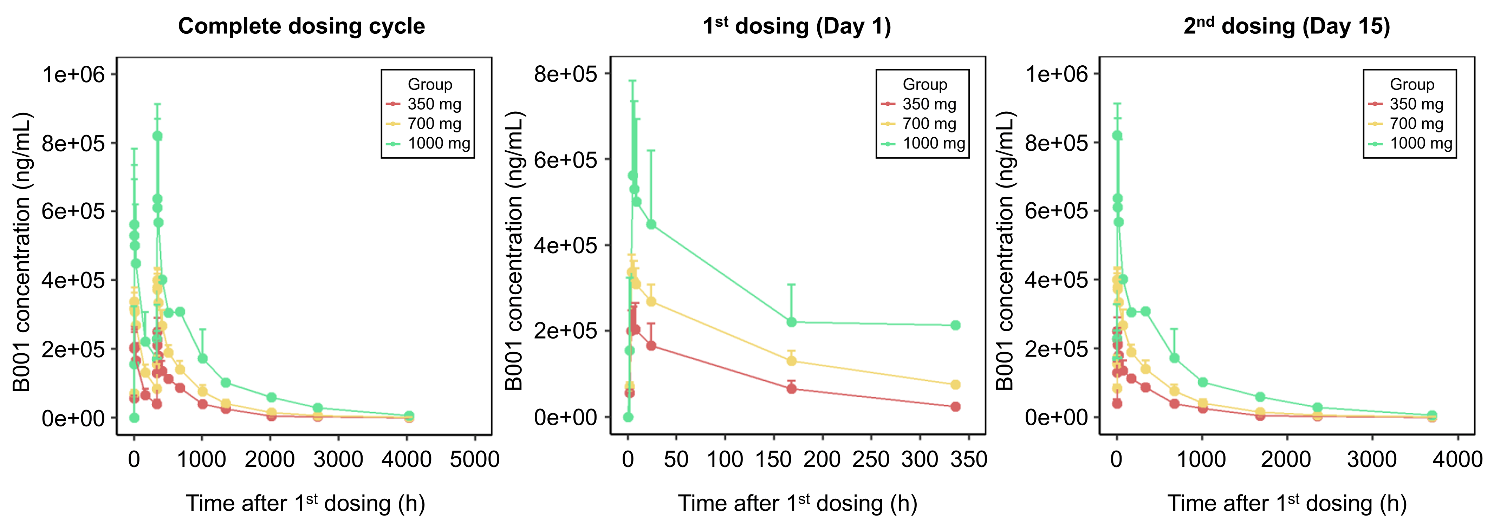


**FIGURE S1.** The serum concentration-time curve of B001 following intravenous infusion in each dose group. Data are presented as mean ± SD.
